# The myeloid mineralocorticoid receptor regulates dermal angiogenesis and inflammation in glucocorticoid‐induced impaired wound healing

**DOI:** 10.1111/bph.15932

**Published:** 2022-09-02

**Authors:** Van Tuan Nguyen, Qui Trung Ngo, Roberto Palacios Ramirez, Toshifumi Nakamura, Nicolette Farman, Sélim Aractingi, Frederic Jaisser

**Affiliations:** ^1^ INSERM, UMRS 1138, Centre de Recherche des Cordeliers Sorbonne Université, Université Paris Cité Paris France; ^2^ Department of Basic Science Thai Nguyen University of Agriculture and Forestry Thainguyen Vietnam; ^3^ Laboratory of Cutaneous Biology, INSERM U1016, Cochin Institute Université Paris Cité Paris France; ^4^ Department of Dermatology Cochin Hospital Paris France

**Keywords:** healing, macrophages, mineralocorticoid, skin

## Abstract

**Background and Purpose:**

Delayed wound healing is among the deleterious consequences of over‐activation of the mineralocorticoid receptor (MR) induced by topical dermocorticoids. The role of dermal inflammation and angiogenesis in the benefits of MR blockade is unknown.

**Experimental Approach:**

Skin wounds were made on C57Bl6 mice after topical pretreatment with the dermocorticoid clobetasol. The impact of topical MR blockade by canrenoate on inflammation, angiogenesis, and the wound macrophage phenotype was analysed 5 days post‐wounding. Similar experiments were conducted on mice with genetic deletion of the MR in myeloid cells.

**Key Results:**

Topical inhibition of the MR with canrenoate improved delayed wound healing through the resolution of prolonged inflammation in glucocorticoid‐pretreated mouse skin. This effect was associated with a higher ratio of anti‐inflammatory macrophages versus pro‐inflammatory macrophages in wounds treated by canrenoate. Furthermore, MR blockade led to upregulated expression of pro‐angiogenic factors and improved impaired angiogenesis in wounds of glucocorticoid‐pretreated skin. Finally, deletion of MR expression by myeloid cells reproduced the benefits of topical pharmacological MR blockade.

**Conclusion and Implications:**

Topical MR antagonism facilitates the switching of macrophages towards an anti‐inflammatory phenotype, which improves prolonged inflammation and induces angiogenesis to accelerate wound healing delayed by glucocorticoid treatment.

## Graphical abstract

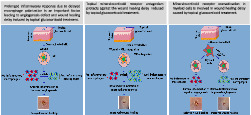

**Abbreviations**
GC: glucocorticoid
GR: glucocorticoid receptor
MFI: mean fluorescence intensity
MR: mineralocorticoid receptor
MRA: mineralocorticoid receptor antagonist

**What is already known**

- Delayed wound re‐epithelialization is a major deleterious effect of topical glucocorticoid treatment.
- Topical mineralocorticoid receptor (MR) antagonism protects against the wound healing delay induced by glucocorticoids.

**What this study adds**

- Topical MR antagonism prevents angiogenesis defect associated to wound healing delay induced by topical glucocorticoids.
- Mineralocorticoid receptor overactivation in myeloid cells is involved in wound healing delay caused by glucocorticoids.

**What is the clinical significance**

- We suggest a therapeutic approach for improving the wound healing defect induced by topical glucocorticoids.

## 1 INTRODUCTION

Cutaneous homeostasis allows the skin to maintain a protective barrier for the organism. As the skin is an interface tissue, continuous wound healing contributes to preserve unaltered cutaneous homeostasis. Wound healing of the skin is a complex phenomenon that is orchestrated by a complex interplay between inflammatory, proliferative, and biological remodelling processes. During the inflammatory phase, macrophages play a central role in producing cytokines, chemokines, growth and angiogenic factors, and metabolites that stimulate cell proliferation and the synthesis of materials necessary for the regeneration of tissue (Gurtner et al., 2008; Hesketh et al., 2017; Raziyeva et al., 2021). Importantly, macrophages express differential phenotypes and activities according to the stage of the healing process. Pro‐inflammatory macrophages are recruited into the wound area at the early stage. They express or secrete various pro‐inflammatory factors, such as TNF‐α, IL1β, MMP9, iNOS and MCP1, to kill pathogens and promote debridement of the altered matrix and the dead cells/tissue. At the later phase of the inflammatory response, anti‐inflammatory macrophages are recruited. They express or secrete anti‐inflammatory factors, including IL10, ARG1,and Fizz1, to resolve inflammation and produce growth factors for tissue regeneration (Aitcheson et al., 2021; Hutami et al., 2021; Murray et al., 2014; Ramalho et al., 2018; Zubair & Ahmad, 2019). The balance and progressive switch between these populations are pivotal for the normal healing process (Larouche et al., 2018; Mahdavian Delavary et al., 2011).

Topical or oral glucocorticoids (GCs) are widely used drugs that induce, sometimes severe, delayed wound healing. GCs induce their biological activity either through binding to the glucocorticoid receptor (GR or NR3C1) but also through binding and activating the mineralocorticoid receptor (NR3C2 or MR). We previously reported that the MR in skin is involved in adverse skin effects, including GC‐induced epidermal atrophy and GC‐ and diabetes‐induced delayed wound closure (Farman & Rafestin‐Oblin, 2001; Jaisser & Farman, 2016).

As GCs are responsible for delayed healing and alterations of macrophage activity, we investigated whether activation of the MR by GC could impair the acute inflammatory response and angiogenesis, thus participating in GC‐induced delayed dermal wound healing, in addition to delayed re‐epithelialization of the wound. We previously reported that pretreatment with clobetasol delays wound healing and that topical MR antagonism with canrenoate improves it (Nguyen et al., 2016). However, the underlying mechanisms are not yet fully understood. We hypothesized that topically applied MR blockers may have a positive impact on inflammation and dermal angiogenesis to accelerate wound healing following GC pretreatment of the skin. Moreover, we identified a critical role of the MR expressed in myeloid cells in this process.

## 2 METHODS

### 2.1 Animal studies

The experimentations were carried out following the rules for laboratory animals care and protection as detailed in Directive 2010/63/EU and revised through Directive 86/609/EEC. Such animal experimentation protocols have been approved by the Darwin Ethical Committee of Pierre et Marie Curie University and the French Ministry of Research (APAFIS#4438‐2015092514508030 v7). Animal studies are reported in compliance with the ARRIVE guidelines (Percie du Sert et al., 2020a) and with the recommendations made by the *British Journal of Pharmacology* (Lilley, Stanford et al., 2020). Randomization and blinded analysis for data analysis were used. The animals were housed at the CEF (Centre d'Explorations Fonctionnelles of the Cordeliers Research Center, Agreement no. B75‐06‐12) in controlled conditions for temperature, light/dark cycle and food. At this stage, the influence of sex has not yet been investigated. We, therefore, only conducted the analysis with female mice. Female C57Bl/6 mice (Janvier Laboratories, France) were used for pharmacological experiments. Female mice in which the MR was knocked out in myeloid cells (LysCre‐MR KO) were obtained through the mating of MR^f/f^ floxed mice (Berger et al., 2006) with the LysM/Cre transgenic mice that express the Cre recombinase under the control of a LysM promoter (Clausen et al., 1999) (The Jackson Laboratory, USA).

### 2.2 Reagents and antibodies

The reagents that have been used in this study were supplied by Sigma‐Aldrich (Saint‐Quentin Fallavier, France). Clobetasol was chosen as it is a potent topical steroid widely used in dermatology for various skin diseases that is able to induce skin atrophy and healing delay.

The following antibodies were used for immunofluorescence staining: rat anti‐mouse F4.80 (1:250; Abcam, Cambridge, UK), rat anti‐Gr‐1 (1:250; eBioscience, Villebon‐sur‐Yvette, France), rat anti‐CD3 (1:250; Biolegend, Paris, France), and rat anti‐mouse CD31 (1:200; BD Biosciences, Le Pont de Claix, France), rabbit anti‐K14 (1:500; Biolegend). The sections were afterwards counterstained with 4,6‐diamidino‐2‐phenylindole 0.3 μg·ml^−1^ (Sigma‐Aldrich, Saint‐Quentin‐Fallavier, France). Flow cytometry analysis was performed using PE/Cy7‐conjugated rat anti‐F4/80 (1:200, eBioscience), FITC‐conjugated rat anti‐CD31 (1:100, Biolegend), APC‐conjugated rat anti‐CD45 (1:1000; BD Bioscience), APC/Cy7‐conjugated rat anti CD11b (1:100; Biolegend), PE‐conjugated rat anti‐Ly6C (1:100, Biolegend), and FITC‐conjugated rat anti‐CD206 (1:100; Biolegend). The immuno‐related procedures used comply with the recommendations made by the *British Journal of Pharmacology* (Alexander et al., 2018).

### 2.3 Excisional wound experimentation in mice

Excisional wounds were carried out in C57BL6 mice and/or LysCre‐MR KO mice with or without topical pretreatment once a day with 125 μg clobetasol (Clarelux, 500 μg·g^−1^, GlaxoSmithKline laboratory, Rueil‐Malmaison, France) on the back for 10 days before wounding. Mice were initially anaesthetized with 4.9% isofluorane (Baxter, Newbury, UK) at 300 ml/min ambient air flow, which was reduced to 2% for maintenance. After shaving, we selected the dorsal areas featured by typical pink telogen stage colour. Excisional 6‐mm diameter wounds were generated on the back of the mice using a disposable device (Kai Europe GmbH, Solingen, Germany). No dressings were applied on the wounds. Treatment consisted in twice daily applications on the wound surface of 30 μl of the MR antagonist potassium canrenoate [0.5 mM]) or PBS until killing (Day 5). Photographs of the mouse backs with wounds were done at various timepoints after wounding using a digital camera (Sony CybershotH 10.1‐megapixel DSC‐W180). The wound surface was calculated as the percentage of unhealed area/initial wound area at Days 3 and 5.

### 2.4 Skin preparation and immunofluorescence staining

Wound specimens were collected and either snap‐frozen in liquid nitrogen, then stored at −80°C, or fixed in 4% formaldehyde for 24 h at 4°C; 6‐μm OCT‐embedded cryosections were obtained. Sections were placed with 2% BSA/PBS for blocking nonspecific binding, then incubated with primary antibodies either during 2 h at room temperature or overnight at 4°C. Alexa Fluor 488 and Alexa Fluor 555 were then used as conjugated secondary antibodies (1:1000) (Invitrogen, Villebon‐sur‐Yvette, France). Samples were examined and photographed using a ZEISS axio scan Z1 (Carl Zeiss, Jena, Germany). F4.80, Gr‐1, and CD3‐labelled cells were expressed as a percentage of total cells (Michalczyk et al., 2018). The surface of CD31^+^ blood vessels was measured and expressed as a ratio of total granulation tissue surface.

### 2.5 Fluorescence activated cell sorting (FACS) analysis

After removal of the intact normal tissue, skin wound specimens were harvested at Day 5 under isoflurane anaesthesia, cut into small pieces and digested with 0.1 mg·ml^−1^ Liberase DH (Roche, Mannheim, Germany) and 10 mg·ml^−1^ DNase I (Sigma) in DMEM, 2% FBS, with continuous stirring at 3°C, three times for 10 minutes. Cell suspensions were prepared following classical techniques (Adamson et al., 2016; Olingy et al., 2017). After each incubation, tissue pieces were mechanically dissociated by repetitive pipetting through a 1 ml micropipette tip. After inactivation of enzymes by DMEM, 10% FBS, the supernatant was filtered through 100 and 40 μm cell strainers, subsequently. One million cells were suspended in the FACS solution (1% FBS/PBS) blocked with anti‐mouse CD16/CD32, then incubated with a combination of fluorescently‐conjugated antibodies in the dark for 1 h at 4°C followed by incubation Then, these cells were washed and placed in the FACS buffer. Flow cytometry was carried out on a Becton Dickinson LSR II cytometer (BD Pharmingen). The FlowJo software (TreeStar Inc., USA) was used to analyse the results. The populations of macrophages and endothelial cells were given as the percentages in relation of total living cells. Similarly, pro‐inflammatory M1 and anti‐inflammatory M2 macrophages subpopulations were expressed as percentages related to total macrophage numbers. A specific marker expression is reported as the geometric mean of conjugated fluorescence intensity (MFI).

### 2.6 RNA extraction and real‐time reverse transcription‐PCR (qRT‐PCR)

Total RNA was extracted from wounded skin using TRIzol reagent (Invitrogen) and the RNeasy Mini kit from Qiagen (Qiagen, Les Ulis, France) followed the manufacturer's instructions. The SuperScript™ II First‐Strand Synthesis kit (Thermo Scientific, France) was used to synthesize cDNA from 1 μg of total RNA. Real‐time PCR was performed with 2 ng cDNA as a template in a final volume of 12 μl using iQ™ SYBR® Green supermix (Bio‐Rad, Marnes‐la‐Coquette, France) and a CFX384 Real‐Time PCR Detection System (Bio‐Rad). All samples were analysed in duplicate. The expression of each target gene was normalized to the levels of two reference house‐keeping genes (GAPDH and beta‐actin). We designed the primers (see the list in supplementary table) that were ordered from Eurogentec (Seraing, Belgium).

### 2.7 Data and statistical analysis

Our data comply with BJP recommendations and requirements on experimental design and analysis (Curtis et al., 2018). The statistical analysis was conducted only when the group sizes were at least n = 5. The group sizes correspond to the number of independent values. Statistical analyses were performed using Graphpad Prism software version 6.0. Results are expressed as the means ± SEM. Differences between the means of two groups were assessed using the non‐parametric Mann–Whitney tests. Differences between multiple groups were analysed by one‐way ANOVA and two‐way ANOVA followed by the Newman–Keuls Multiple Comparison test. Post‐hoc tests were run only if F achieved P<0.05 and there was no significant variance inhomogeneity.

### 2.8 Nomenclature of targets and ligands

Key protein targets and ligands in this article are hyperlinked to corresponding entries in the IUPHAR/BPS Guide to PHARMACOLOGY https://www.guidetopharmacology.org, and are permanently archived in the Concise Guide to PHARMACOLOGY 2021/22 (Alexander, Cidlowski et al., 2021; Alexander, Fabbro et al., 2021; Alexander, Mathie et al., 2021).

## 3 RESULTS

### 3.1 Inhibition of the MR rescues glucocorticoid‐induced impairment of wound angiogenesis in mice

**FIGURE 1:**
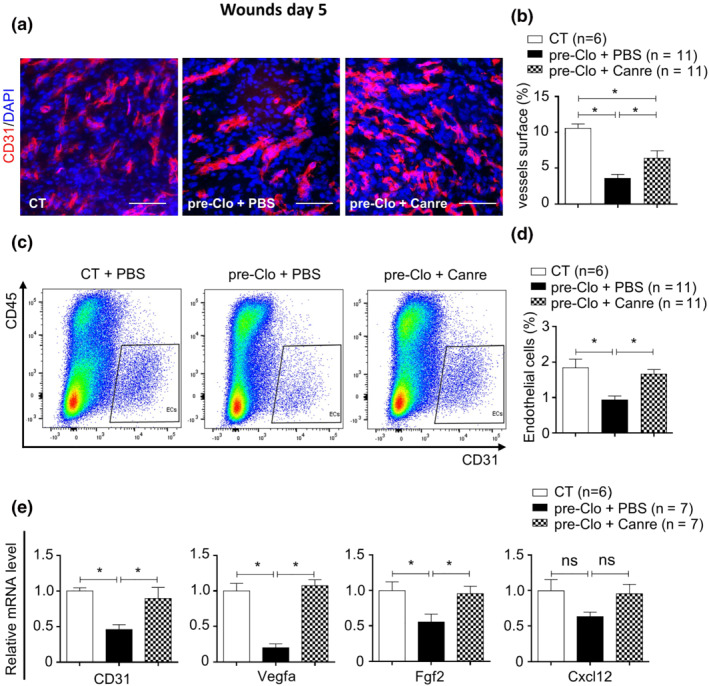
Canrenoate rescues impaired wound angiogenesis in clobetasol‐pretreated skin. (a) Images of wound sections doubled‐stained for CD31 (red) and DAPI (blue), showing neo‐microvessels in the wound beds of mice pretreated with clobetasol and (b) quantification of CD31^+^ microvessels in wounds after treatment with canrenoate or PBS. (c) Dot plot of FACS analysis of wound cells for the quantification of CD45‐CD31^+^ (dD) endothelial cells in the wounds of mice pretreated with clobetasol after treatment with canrenoate or PBS. (e) Pro‐angiogenic mRNA levels in whole wounded skin from clobetasol‐pretreated mice treated with canrenoate or PBS, relative to wounds of CT mice treated with PBS. Data represent mean ± SEM; n = the number of mice per group from three experimental series. One‐way ANOVA followed by the Newman–Keuls multiple comparison test. *P < 0.05, ns = not significant. ANOVA, analysis of variance; Canre, canrenoate; CT, control; FACS, fluorescence activated cell sorting; PBS, phosphate buffered saline; SEM, standard error of the mean; pre, pretreated; Clo, clobetasol. Scale bar = 100 μm.

As previously published (Nguyen et al., 2016), topical treatment of wounds with the MR antagonist canrenoate reversed the clobetasol induced wound healing delay in mouse skin (Figure S1). Because dermal angiogenesis plays a major role in the granulation tissue to promote wound healing, we investigated the effect of MR antagonism on wound angiogenesis in mice pretreated with a powerful topical steroid, namely Clo (clobetasol gel). The blood vessel density was quantified in the granulation tissue area of wound sections 5 days after wounding. The percentage of CD31^+^ blood vessel area was lower in wounds of Clo‐pretreated skin than those of control skin, indicating a defect in angiogenesis induced by the clobetasol pre‐treatment (Figure 1a,b). Such impaired angiogenesis was partially improved in the wounds treated locally with a reference MR antagonist (canrenoate potassium). Blood‐vessel density in the wound beds of Clo‐pretreated skin improved by 77% after 5 days of canrenoate treatment (Figure 1a,b). FACS analysis of wound specimens showed a higher number of CD45^−^CD31^+^ endothelial cells in wounded skin treated with canrenoate (Figure 1c,d). We also observed lower CD31 mRNA levels in the wounds of Clo‐pretreated skin. The observed reduction was reversed by canrenoate treatment (Figure 1e). Consistent with this effect canrenoate treatment also reversed the mRNA repression of several pro‐angiogenic factors (Vegfa, Fgf2, and Cxcl12) in wounds of Clo‐pretreated skin (Figure 1e). Thus, pretreatment of skin with clobetasol led to impaired dermal wound angiogenesis, which was reversed by topical MR antagonism.

### 3.2 MR antagonism induces anti‐inflammatory macrophage polarization in glucocorticoid‐pretreated skin

**FIGURE 2:**
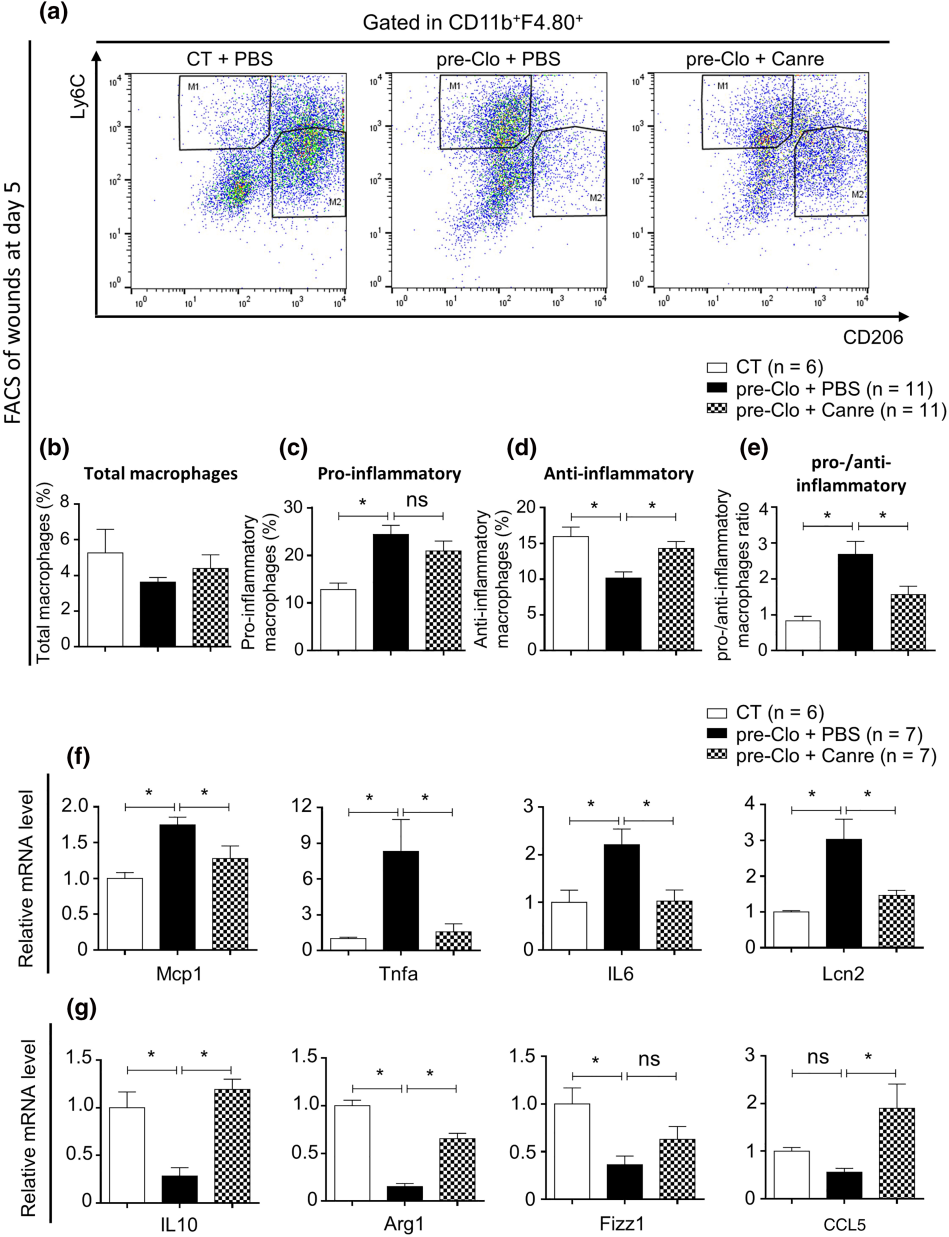
Canrenoate enhances polarization of pro‐inflammatory to anti‐inflammatory macrophages to regulate the inflammatory response. (a) Dot plot of FACS analysis of wound cells allowing quantification of (c) CD11b^+^ F4.80^+^ Ly6C^hi^ pro‐inflammatory and (d) CD11b^+^ F4.80^+^Ly6C^low^ anti‐inflammatory macrophages in the wounds of clobetasol‐pretreated mice after treatment with canrenoate or PBS as the percentage of (b) total CD11b^+^ F4.80^+^ macrophages and the (e) pro‐/anti‐inflammatory macrophage ratio. Cytokine mRNA levels were analysed by quantitative RT‐PCR. (f) Pro‐inflammatory cytokine mRNA levels in the wounds of Clo‐pretreated mice treated with canrenoate or PBS relative to those of CT wounds treated with PBS. mRNA levels of anti‐inflammatory cytokines in the (g) wounds of clobetasol‐pretreated mice treated with canrenoate or PBS relative to those of CT wounds treated with PBS. Data represent mean ± SEM; n = the number of mice per group from three experimental series. One‐way ANOVA followed by the Newman–Keuls multiple comparison test. *P < 0.05, ns = not significant. ANOVA, analysis of variance; Canre, canrenoate; CT, control; FACS, fluorescence activated cell sorting; PBS, phosphate buffered saline; RT‐PCR, reverse transcriptase PCR; SEM, standard error of the mean; pre, pretreated; Clo, clobetasol

The polarization of activated pro‐inflammatory macrophages towards alternative anti‐inflammatory macrophages is an important step during the wound healing process. A failure of this switch leads to the prolongation of inflammation with impaired wound healing (Guo et al., 2016; Okizaki et al., 2015; Yan et al., 2018). Here, we asked whether topical MR blockade with canrenoate provides a beneficial effect in the switching of activated pro‐inflammatory macrophages towards the anti‐inflammatory macrophage population. Five days post‐wounding, Clo‐pretreated skin showed more pro‐inflammatory macrophages (Ly6C^hi^CD206^low^) associated with fewer anti‐inflammatory macrophages (Ly6C^low^CD206^hi^) as compared with control skin (Figure 2a,c,d). Importantly, the abnormal polarization of anti‐inflammatory macrophages in the wounds of Clo‐pretreated skin was mostly normalized by topical canrenoate treatment. Although the total number of macrophages was not significantly modified (Figure 2b, Figure S2A‐B), canrenoate treatment increased the proportion of anti‐inflammatory macrophages and reduced the sustained presence of pro‐inflammatory macrophages in the wounds of Clo‐pretreated skin (Figure 2a–d), leading to a decrease in the pro‐/anti‐inflammatory macrophage ratio (Figure 2e). In accordance with the observed change in the macrophage switch, clobetasol pretreatment was associated with the overexpression of various proinflammatory genes, such as Tnf‐a, Mcp‐1, Il‐6, and Lcn‐2 (Figure 2f) and the repression of those for anti‐inflammatory factors, including Il‐10, Arg‐1, Fizz1, and Rantes (CCL5) (Figure 2g), in wounded skin at Day 5. Conversely, MR blockade with canrenoate reversed these modifications (Figure 2f,g).

Additionally, we also evaluated the effects of MR antagonism on recruitment of neutrophils and T cells into wound sites. The total neutrophils and T cells in wounds at Day 5 were quantified by immunostaining of wound sections with the antibodies anti Gr‐1 and CD3, respectively. The results did not show any significant difference of Gr‐1^+^ neutrophils nor CD3^+^ T cells in wounds treated with canrenoate compared with non‐treated wounds (Figure S2C‐D, E‐F).

These observations suggest that MR activation by GCs impairs the beneficial switch of pro‐inflammatory macrophages towards anti‐inflammatory macrophages and that topical MR antagonism prevents this effect, leading to resolution of the prolonged inflammation and the induction of dermal angiogenesis, thus promoting wound healing.

### 3.3 MR activation in myeloid cells is involved in glucocorticoid‐induced delayed wound healing

**FIGURE 3:**
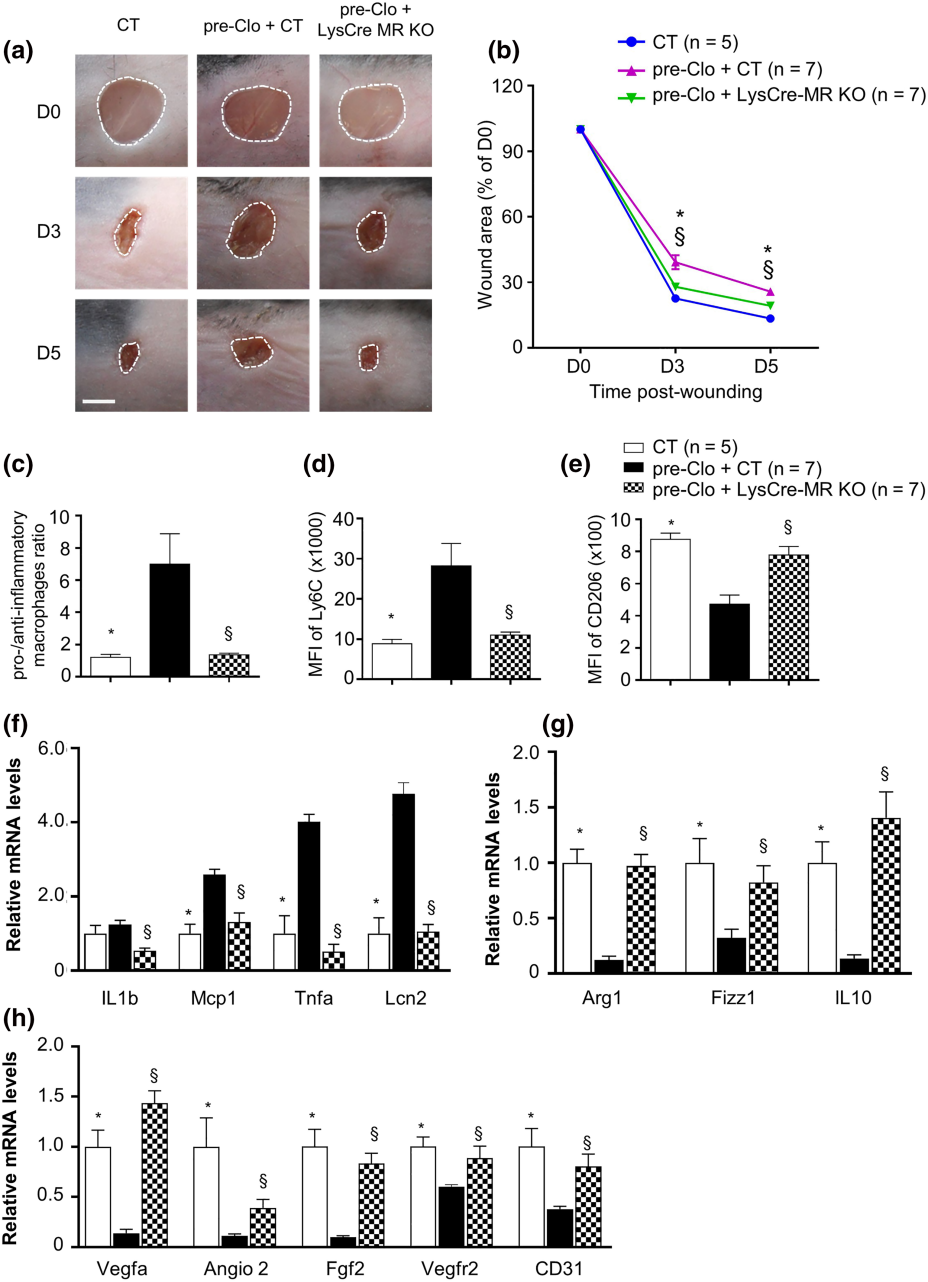
Mineralocorticoid receptor (MR) deficiency in myeloid cells prevents wound healing delay induced by glucocorticoids. (a) Images and (b) quantification of the wound area from control (CT) and Lyscre‐MR KO mice with or without clobetasol pretreatment, at different times post‐wounding. (c) Pro‐/anti‐inflammatory macrophage ratio and (d, e) mean of fluorescence intensity of Ly6C and CD206 quantified by FACS analysis in the macrophage population of wounds at Day 5. (f–h) mRNA levels of pro‐inflammatory, anti‐inflammatory, and angiogenic factors expressed in whole wounded skin. Data represent mean ± SEM; n = the number of mice per group from two experimental series. (b) Two‐way ANOVA, (c–h) one‐way ANOVA, followed by the Newman–Keuls multiple comparison test. *P < 0.05 for CT + Clo versus CT*. §P* < 0.05 for CT + Clo versus LysCre MR KO + Clo. ANOVA, analysis of variance; SEM, standard error of the mean; MR, mineralocorticoid receptor; MFI, mean fluorescence intensity; CT, control; FACS, fluorescence activated cell sorting; PBS, phosphate buffered saline; Canre, potassium canrenoate; KO, knockout; pre, pretreated; Clo, clobetasol; D0, Day 0; D3, Day 3; D5, Day 5. Scale bar = 100 μm

We have shown that topical MR antagonism improves the altered macrophage switching induced by GCs, orienting it back towards the anti‐inflammatory phenotype. We next investigated the specific contribution of the myeloid MR in delayed wound healing of GC‐treated skin. We used a mouse model with a MR gene inactivation in myeloid cells (LysCre‐MR KO) in which endogenous MR expression is drastically reduced in macrophages (Figure S3). After 10 days of clobetasol treatment, wounds were generated on the backs of control MR f/f (CT) and LysCre‐MR KO mice. As expected, the wound closure of the Clo‐pretreated skin was significant delayed as compared with that of control mice. In contrast, the delay in wound healing induced by clobetasol was fully prevented in LysCre‐MR KO mice (Figure 3a,b). FACS analysis of wound specimens showed that myeloid MR deficiency led to a reduction in the pro‐inflammatory macrophage population and the induction of anti‐inflammatory macrophages, as estimated by the mean fluorescence intensity (MFI) of the pro‐inflammatory macrophage marker Ly6C (Figure 3d) and the anti‐inflammatory macrophage marker CD206 (Figure 3e), leading to normalization of the pro‐inflammatory/anti‐inflammatory ratio in the wounds of Clo‐pretreated LysCre‐MR KO mice relative to control mice (Figure 3c). In accordance with this observation, the over expression of various proinflammatory genes, such as Tnf‐a, Mcp‐1, Il‐6, and Lcn‐2 (Figure 3f), and the repression of anti‐inflammatory factors, including Il‐10, Arg‐1, Fizz1, and CCL5, was fully blunted in the wounds of Clo‐pretreated LysCre‐MR KO mice (Figure 3g). Importantly, myeloid‐specific MR deficiency prevented the repression of the expression of various pro‐angiogenic genes (Figure 3h) and impairment of wound angiogenesis (Figure S4E‐F) induced by clobetasol treatment, mimicking the effect of topical MR antagonism.

Furthermore, we investigated whether topical treatment with canrenoate could bring any additional effect on wound healing of these LysCre‐MR KO mice. Our results showed that topical application of canrenoate on the wounds did not bring any significant additive effect on wound closure of LysCre‐MR KO mice (Figure S4A‐B) nor wound re‐epithelialization (Figure S4C‐D) or angiogenesis (Figure S4E‐F) (immunostaining quantification of wound sections at Day 5). Altogether, these results reveal the critical implication of Myeloid MR in wound healing delay induced by glucocorticoid.

## 4 DISCUSSION

GCs are widely used drugs, either systemically or for repeated topical application to skin and mucous membranes. GCs induce changes in normal treated skin that are visible to the naked eye and by histology. They consist of atrophy, telangiectasia, an increase in the number of hair follicles, increased susceptibility to infections, and changes in pigmentation and sebum secretion (Hengge et al., 2006). Long‐term treatment with steroids, either orally or locally, induces healing abnormalities that are susceptible to lead to chronic wounds (Jozic et al., 2017). Importantly, such wounds sometimes result in severe consequences, such as infections, pain, ankylosis, and even carcinogenesis.

Delayed wound healing appears to be at least partially linked to over‐activation of the MR induced by topical dermocorticoids (Nguyen et al., 2016). Herein, we investigated the role of the GC‐MR pathway in the regulation of dermal inflammation and angiogenesis during wound healing. We show that topical inhibition of the MR provides a clear benefit for impaired wound healing induced by topical GC pretreatment through the improvement of dermal angiogenesis and wound closure. This improvement results both from a macrophage switch that allows resolution of the inflammation as well as enhancement of angiogenesis through secretion of proangiogenic peptides.

Our results are original because they suggest the importance of the expression of MR in myeloid cells in the GC induced delayed wound healing rather than in keratinocytes. In various organs, macrophages are also major players of the healing process after tissue damage (Brancato & Albina, 2011; Fraccarollo et al., 2019; Lucas et al., 2010; Rahmani et al., 2018). To adapt to the stage of tissue repair, macrophages express different phenotypes and functional activities. (Boniakowski et al., 2017; Fathy et al., 2014). The balance in switching of such macrophage phenotypes is critical for the progression of wound healing. Accordingly, a prolonged persistence of pro‐inflammatory macrophages with over‐production of pro‐inflammatory cytokines will impair healing (Boniakowski et al., 2017; Larouche et al., 2018; Leal et al., 2015; Okizaki et al., 2015). In this study, we demonstrated that topical MR antagonism rescues the delayed shift towards the anti‐inflammatory phenotype of macrophages.

We previously reported similar findings in delayed wound healing of diabetic skin (Nguyen et al., 2020). Importantly, we previously demonstrated a deleterious impact of MR over‐activation in switching macrophage phenotype during the repair process after renal damage, contributing to prolonged renal injury (Barrera‐Chimal et al., 2018). In cardiac and aortic tissue, it has been demonstrated that MR deficiency in myeloid cells could prevent the proinflammatory profile of macrophages which protect against cardiac inflammation, fibrosis, and vascular damage (Shen et al., 2016; Usher et al., 2010). In accordance, Fraccarollo et al. stated that macrophage MR is a crucial regulator of macrophage differentiation and cardiac wound repair after myocardial infarction (Fraccarollo et al., 2019). Altogether, these finding support a potential common mode of action of MR antagonism in controlling macrophage polarization during tissue repair processes. In another view, both glucocorticoid and aldosterone could have effects on multiple types of immune cell including regulation of lymphocytes, dendritic cells, neutrophils (Elenkov, 2004; Hu et al., 2018; Jaisser & Farman, 2016; Tuckermann et al., 2007).

Angiogenesis defects can be due to a prolonged inflammatory response that leads to an impairment in pro‐angiogenic factors production and functional abnormalities of endothelial cells (Edwards et al., 2018; Loomans et al., 2004). Indeed, macrophages secrete several pro‐angiogenic peptides. Here, we found that inhibition of the myeloid MR led to a higher expression of pro‐angiogenic factors, contributing to an improved angiogenesis and enhancement of wound healing delay induced by clobetasol. Targeted MR inactivation in myeloid cells mimicked the effect of the topical MRA canrenoate on the properties of wound macrophages and expression of proangiogenic factors. Overall, these results suggest an important role for myeloid MR signalling in the sustained polarization of macrophages and impaired wound angiogenesis. Of note, the LysM promotor is myeloid‐specific but not macrophage‐specific, i.e. it allows the Cre recombinase expression in neutrophils, monocytes, macrophages and dendritic cells, leading to gene inactivation also in these cell types. In another view, it would be interesting to investigate whether these phenomena can be affected by the MR signalling in other cell types including endothelial cells. Indeed, previous studies reveal that activation of endothelial cell MR impairs angiogenesis‐related gene expression and angiogenic capacity and induces the capillary rarefaction which plays a pivotal role pathophysiology of hypertension and related heart disease (Biwer et al., 2021; Lother et al., 2019). Moreover, Rickard et al. demonstrated that endothelial cell MR signalling contributes in the inflammatory and profibrotic response deoxycorticosterone/salt (Rickard et al., 2014).

These findings provide insights on the impact of MR activation in several cutaneous diseases. Indeed, we previously reported the role of cutaneous MR over‐activation in the deleterious effects induced by topical glucocorticoid treatments both in mice and humans: GC‐induced MR activation impaired the proliferation and activation of epidermal keratinocytes and local skin treatment with MR antagonists led to significant benefits (Maubec et al., 2015; Nguyen et al., 2016). Also, an increase in MR expression in keratinocytes induces atrophy of the mouse epidermis (Sainte Marie et al., 2007), whereas mice in which the MR was inactivated in epithelial cells showed increased keratinocyte proliferation and differentiation (Boix et al., 2016). Deletion of the MR in keratinocytes was shown to alter the homeostasis of all skin layers in aged mice, with reduced dermal thickness and decreased collagen deposition (Bigas et al., 2020). We previously showed that topical MR blockade accelerates skin wound healing in diabetic mice (Nguyen et al., 2020). Biyashev et al. recently reported that the association of vitamin D3 and the MR antagonist spironolactone improves the inflammation shift and therefore enhances wound healing induced by nitrogen mustard exposure (Biyashev et al., 2020). A recent study extended the implication of GC‐induced off‐target MR activation in skin defects associated with psychological stress, acting through an increase in endogenous GC cortisol levels, which alters epidermal barrier function and worsens several common skin diseases, such as atopic dermatitis or psoriasis (Lee et al., 2021). This study showed that MR antagonism improved the deleterious consequences of GC on skin barrier function found in normal human epidermal keratinocytes and reconstructed human epidermis (Lee et al., 2021).

Importantly, we observed in a previous study that Clobetasol pre‐treatment of mouse skin leads to an increased expression in the wound of epithelial sodium channels (ENaC), a target gene downstream of MR activation, that was blunted when the wound was treated with the MR blocker Canrenoate. This effect was similarly observed in diabetic skin (Nguyen et al., 2020). These results favour the view that MR activation does occur in glucocorticoid‐treated epidermis and in diabetic skin, as MR blockade was efficient at reducing Clobetasol (or diabetes‐)‐associated excessive ENaC expression. Of note, the glucocorticoid receptor (GR) is also activated by clobetasol treatment. Therefore, the possibility of a GR/MR heterodimer cannot be excluded. At the present time, it is very difficult to know whether such heterodimers do exist in vivo, more specifically in the skin and in all cell types. Overall, we consider that reducing the MR occupancy by exogenous or endogenous GCs will be responsible for beneficial effects of MR blockade in several situations, such as glucocorticoids treatment, ageing, UV irradiation, diabetic delayed wound healing, and perhaps psychological stress (Hannen et al., 2017; Lee et al., 2021; Nguyen et al., 2016; Stojadinovic et al., 2016).

In summary, our results suggest that persistent GC stimulation of the MR is responsible for prolonged effects that can be prevented by MR blockade. We focused here on a major role of MR signalling in the inflammatory steps as well as impaired vascular changes that develop in dermocorticoid‐induced delayed wound healing in the mouse. These results suggest that topical MR inhibitors could also be effective in similar situations in affected patients. Importantly, we showed that the myeloid MR plays a critical role in promoting local inflammation and that MR antagonism promotes anti‐inflammatory macrophage polarization and dermal angiogenesis, thus promoting tissue repair. Therefore, the use of topical MR antagonists could represent a practical future tool for numbers of patients complaining of delayed healing induced by GC treatments.

## AUTHOR CONTRIBUTIONS

Design: VTN, NF, SA, and FJ; Data Curation and Analysis: VTN, QTN and TN. Funding: SA and FJ; Supervision: NF, SA, and FJ; Writing ‐ Original Draft Preparation: VTN, QTN, NF, SA, and FJ.

## CONFLICT OF INTERESTS

The authors have no conflict of interests to declare.

## DECLARATION OF TRANSPARENCY AND SCIENTIFIC RIGOUR

This Declaration acknowledges that this paper adheres to the principles for transparent reporting and scientific rigour of preclinical research as stated in the *BJP* guidelines for Design and Analysis, Immunochemistry and Animal Experimentation, and as recommended by funding agencies, publishers and other organizations engaged with supporting research.

## Supporting information


**Figure S1.** MR antagonist rescues clobetasol induced wound healing delayed in mouse skin*.*
Photographs (A) and (B), quantification of wound area from mice with and without pretreated (10 days) with clobetasol and treated with Canrenoate (Canre) or PBS at different time points post wounding. Data represent mean ± SEM; n = number of mice per group. Statistics: (B) two‐way ANOVA test followed by the Newman–Keuls Multiple Comparison test. *p < .05 for pre‐Clo + PBS vs CT*. §p < .05* for pre‐Clo + PBS vs pre‐Clo + Canre, ns = not significant. ANOVA, analysis of variance; CT, control; Canre, potassium canrenoate; PBS, phosphate buffered saline; SEM, standard error of the mean; pre, pretreated; Clo, clobetasol; D0, day 0; D3, day 3; D5, day 5. Scale bar = 100 μm*.*
Figure S2. Canrenoate does not modify the recruitment of inflammatory cells in clobetasol pre‐treated skin.
Photographs of wound sections at day 5 post‐wounding labelled with (A) anti‐ F4.80; (C) anti‐ Gr‐1 and (E) anti‐CD3 antibodies, showing neutrophils, macrophages, lymphocyte T cells in granulation tissue of mice pre‐treated clobetasol and (B, D, F) quantification of these cells in granulation tissue of wounds at day 5 after treatment with Canre or PBS. Data present mean ± SEM, n = number of mice per group, from 2 experimental series. Differences between the means
Figure S3. MR mRNA expressed low level in macrophages of LysCre‐MR KO mice model.
The expression level of MR mRNA is decreased in macrophages from the LysCre‐MR KO mice model compared with CT mice. Data present mean ± SEM, n = number of mice per group. Differences between the means of two groups were assessed using a non‐parametric Mann–Whitney test. *p < .05, MR, mineralocorticoid receptor; CT, control; KO, knockout; SEM, standard error of the mean.
Figure S4. Canrenoate does not give an additional effect on wound healing of myeloid mineralocorticoid receptor deficiency mice pretreated with clobetasol.
(A) Images and (B) quantification of the wound area from CT or LysCre‐MR KO mice with or without clobetasol pretreatment before wounding and canrenoate treatment post‐wounding, at the different time points post‐wounding. (C) Photographs of wound sections at day 5 post‐wounding labelled with anti‐K14 antibody (green) and DAPI (blue). (D) Quantification of the length of the neo‐epidermis. (E) Photographs of wound sections stained for CD31 (red), showing neo‐microvessels formed in wound beds. (F) Quantification of CD31 + microvessels surface in the granulation tissue of wounds. Data represent mean ± SEM; n = number of mice per group. Statistics: (B): two‐way ANOVA; (D), (F): one‐way ANOVA test followed by the Newman–Keuls Multiple Comparison test. *p < .05 for CT + Clo vs CT*. §p < .05* for CT + Clo vs LysCre‐MR KO + Clo, *ns = not significant*. ANOVA, analysis of variance; K14, keratin‐14; DAPI, 4,6‐diamidino‐2‐phenylindole; MR, mineralocorticoid receptor; CT, control; Canre, potassium canrenoate; SEM, standard error of the mean; pre, pretreated; Clo, clobetasol; KO, knockout; D0, day 0; D3, day 3; D5, day 5. Scale bar = 100 um

**Table S1.** The sequences of quantitative RT‐PCR primers

## Data Availability

The related data and materials are available for sharing upon request to F.J. The data used to support the findings of this study are available from the corresponding author upon reasonable request. Some data may not be made available because of privacy or ethical restrictions.
