# Supplementary material for: The myeloid mineralocorticoid receptor regulates dermal angiogenesis and inflammation in glucocorticoid‐induced impaired wound healing
Source: Br J Pharmacol. 2022 Sep 2;179(23):5222–32. doi: 10.1111/bph.15932 (PMC9826027; doi:10.1111/bph.15932)
Supplement: Supplementary file 1 — Figure S1. MR antagonist rescues clobetasol induced wound healing delayed in mouse skin. Photographs (A) and (B), quantification of wound area from mice with and without pretreated (10 days) with clobetasol and treated with Canrenoate (Canre) or PBS at different time points post wounding. Data represent mean ± SEM; n = number of mice per group. Statistics: (B) two‐way ANOVA test followed by the Newman–Keuls Multiple Comparison test. *p < .05 for pre‐Clo + PBS vs CT. §p < .05 for pre‐Clo + PBS vs pre‐Clo + Canre, ns = not significant. ANOVA, analysis of variance; CT, control; Canre, potassium canrenoate; PBS, phosphate buffered saline; SEM, standard error of the mean; pre, pretreated; Clo, clobetasol; D0, day 0; D3, day 3; D5, day 5. Scale bar = 100 μm. Figure S2. Canrenoate does not modify the recruitment of inflammatory cells in clobetasol pre‐treated skin. Photographs of wound sections at day 5 post‐wounding labelled with (A) anti‐ F4.80; (C) anti‐ Gr‐1 and (E) anti‐CD3 antibodies, showing neutrophils, macrophages, lymphocyte T cells in granulation tissue of mice pre‐treated clobetasol and (B, D, F) quantification of these cells in granulation tissue of wounds at day 5 after treatment with Canre or PBS. Data present mean ± SEM, n = number of mice per group, from 2 experimental series. Differences between the means Figure S3. MR mRNA expressed low level in macrophages of LysCre‐MR KO mice model. The expression level of MR mRNA is decreased in macrophages from the LysCre‐MR KO mice model compared with CT mice. Data present mean ± SEM, n = number of mice per group. Differences between the means of two groups were assessed using a non‐parametric Mann–Whitney test. *p < .05, MR, mineralocorticoid receptor; CT, control; KO, knockout; SEM, standard error of the mean. Figure S4. Canrenoate does not give an additional effect on wound healing of myeloid mineralocorticoid receptor deficiency mice pretreated with clobetasol. (A) Images and (B) quantification of the wound area [file BPH-179-5222-s001.pdf]

SUPPLEMENTARY FIGURES

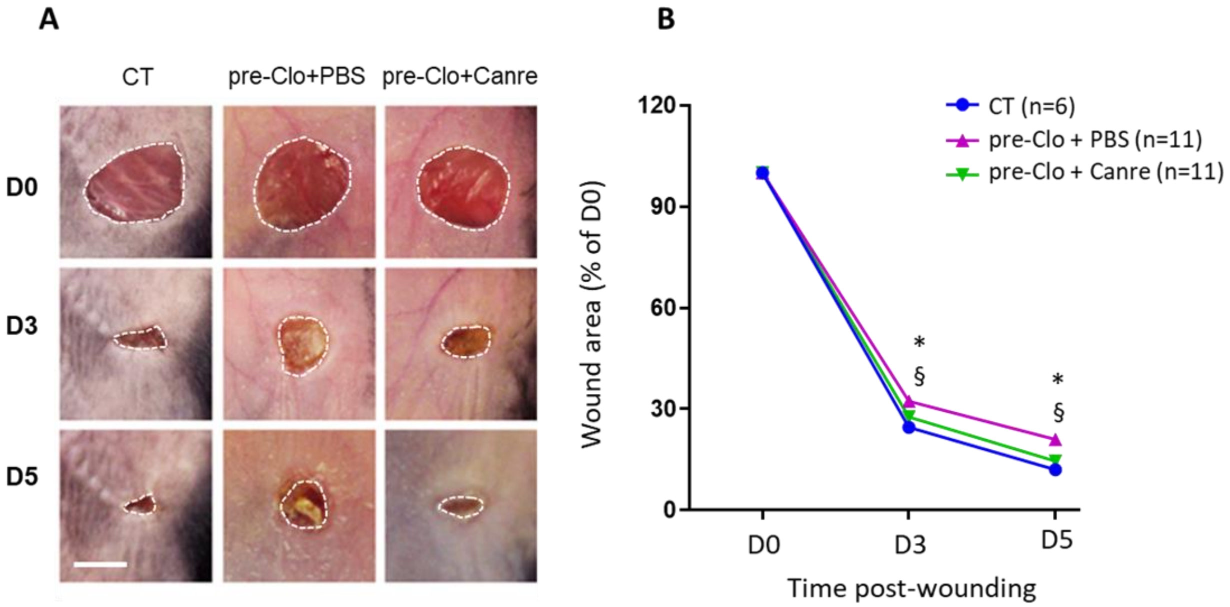

Figure S1. MR antagonist rescues clobetasol induced wound healing delayed in mouse skin.

Photographs (**A**) and (**B**) quantification wound area from mice with and without pretreated 10 days with clobetasol and treated with Canrenoate (Canre) or PBS at different time point post wounding. Data represent mean ± SEM; n= number of mice per group. Statistics: **(B)** two-way ANOVA test followed by the Newman-Keuls Multiple Comparison test. $*p < 0.05$ for pre-Clo + PBS vs CT. $§p < 0.05$ for pre-Clo + PBS vs pre-Clo + Canre, ns = not significant. ANOVA, analysis of variance; CT, control; Canre, potassium canrenoate; PBS, phosphate buffered saline; SEM, standard error of the mean; pre, pretreated; Clo, clobetasol; D0, day 0; D3, day 3; D5, day 5. Scale bar = 100 µm.

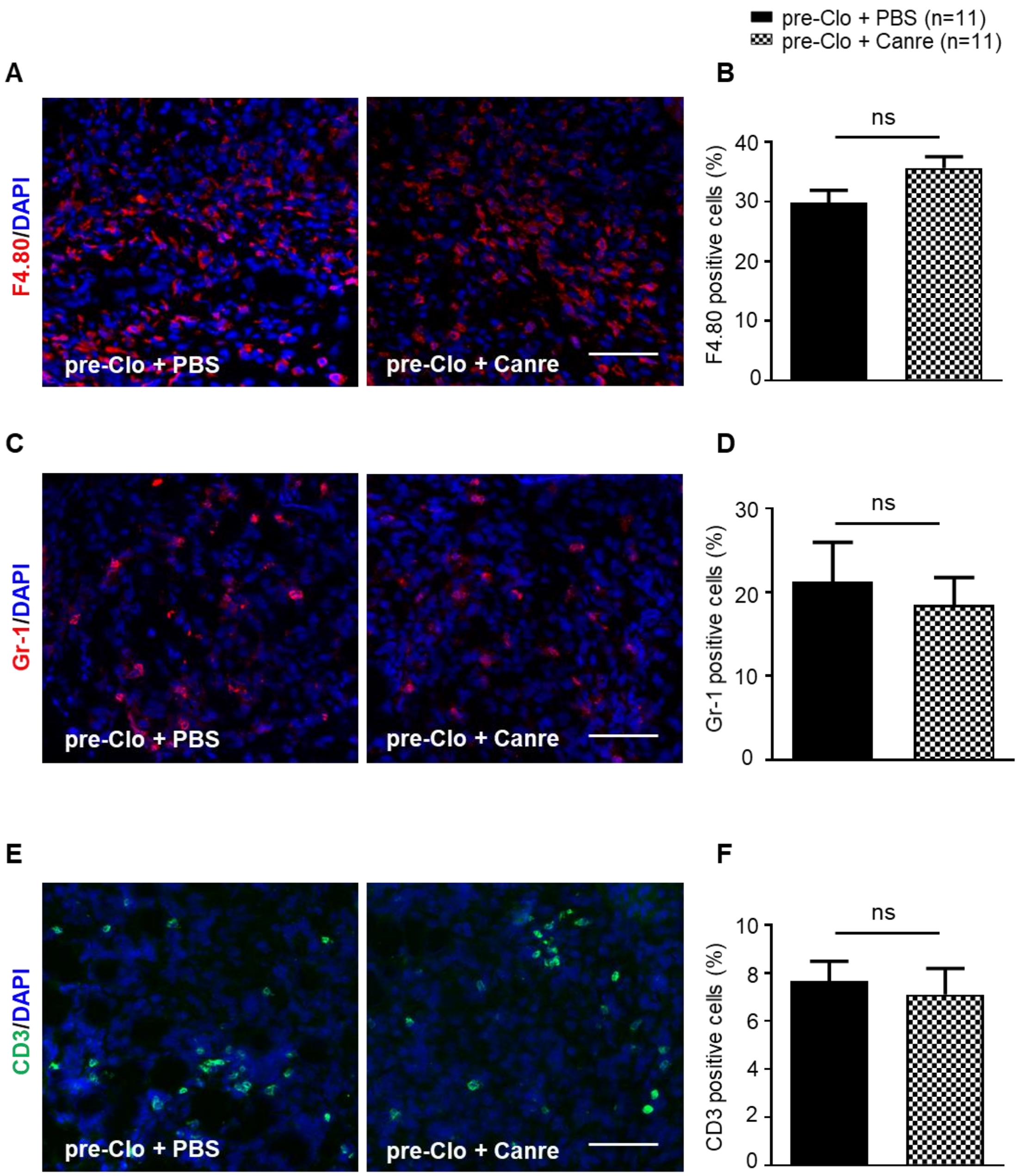

Figure S2. Canrenoate does not modify the recruitment of inflammatory cells in clobetasol pre-treated skin.

Photographs of wound sections at day 5 post-wounding labelled with (**A**) anti- F4.80; (**C**) anti-Gr-1 and (**E**) anti-CD3 antibodies, showing neutrophils, macrophages, lymphocyte T cells in granulation tissue of mice pre-treated clobetasol and (**B, D, F**) quantification of these cells in granulation tissue of wounds at day 5 after treatment with Canre or PBS. Data present mean ± SEM, n= number of mice per group, from 2 experimental series. Differences between the means

of two groups were assessed using a non-parametric Mann-Whitney test. *ns: not significant*. PBS, phosphate buffered saline; Canre, potassium canrenoate; DAPI, 4,6-diamidino-2-phenylindole; SEM, standard error of the mean; pre, pretreated; Clo, clobetasol. Scale bar = 100 µm

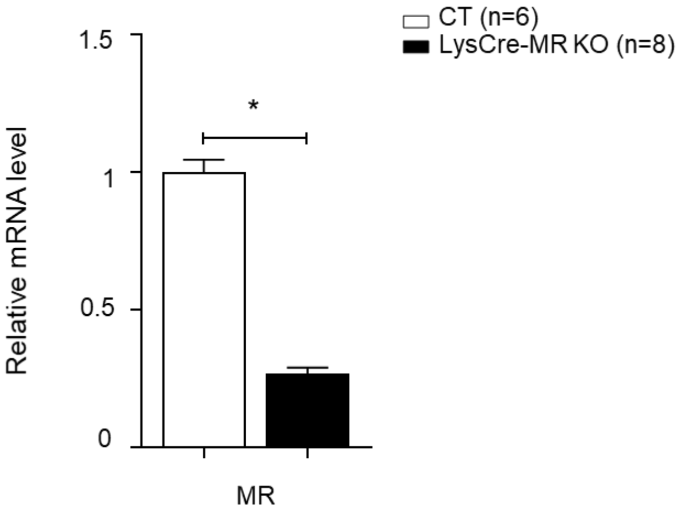

Figure S3. MR mRNA expressed low level in macrophages of LysCre-MR KO mice model. The expression level of MR mRNA is decreased in macrophages from the LysCre-MR KO mice model compared with CT mice. Data present mean ± SEM, n = number of mice per group. Differences between the means of two groups were assessed using a non-parametric Mann-Whitney test. $*p < 0.05$, MR, mineralocorticoid receptor; CT, control; KO, knockout; SEM, standard error of the mean.

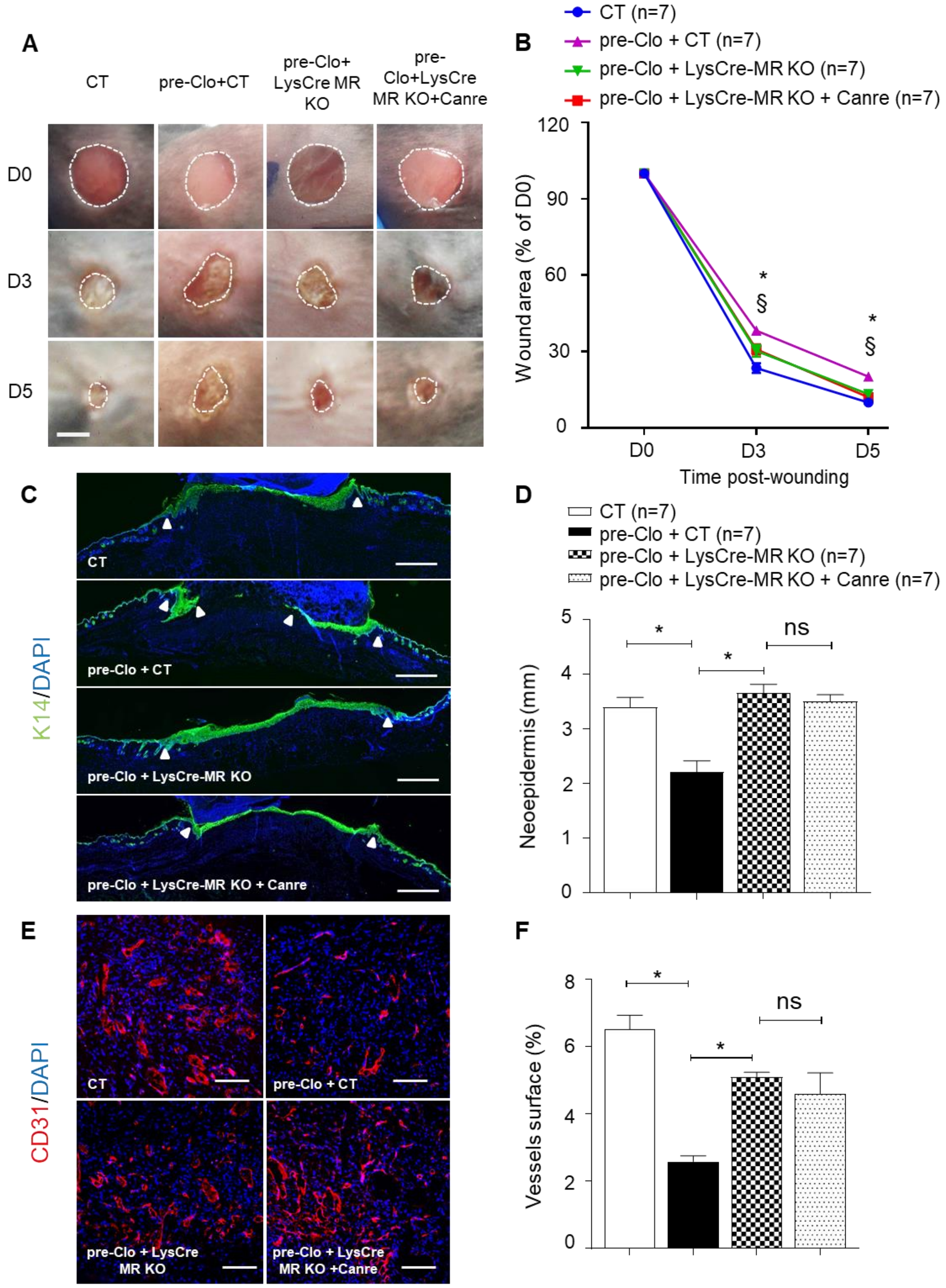

Figure S4. Canrenoate does not give additional effect on wound healing of myeloid mineralocorticoid receptor deficiency mice pretreated with clobetasol.

(**A**) Images and (**B**) quantification of the wound area from CT or LysCre-MR KO mice with or without clobetasol pretreatment before wounding and canrenoate treatment post-wounding, at

the different time points post-wounding. (**C**) Photographs of wound sections at day 5 post-wounding labeled with anti-K14 antibody (green) and DAPI (blue). (**D**) Quantification of the length of the neo-epidermis. (**E**) Photographs of wound sections stained for CD31 (red), showing neo-microvessels formed in wound beds. (**F**) Quantification of $CD31^+$ microvessels surface in the granulation tissue of wounds. Data represent mean ± SEM; n= number of mice per group. Statistics: (**B)**: two-way ANOVA; **(D)**, **(F)**: one-way ANOVA test followed by the Newman-Keuls Multiple Comparison test. *$*p < 0.05$* for CT + Clo vs CT. *§$p < 0.05$* for CT + Clo vs LysCre-MR KO + Clo, *ns = not significant*. ANOVA, analysis of variance; K14, keratin-14; DAPI, 4,6-diamidino-2-phenylindole; MR, mineralocorticoid receptor; CT, control; Canre, potassium canrenoate; SEM, standard error of the mean; pre, pretreated; Clo, clobetasol; KO, knockout; D0, day 0; D3, day 3; D5, day 5. Scale bar = 100 um
