# Supplementary material for: The myeloid mineralocorticoid receptor regulates dermal angiogenesis and inflammation in glucocorticoid‐induced impaired wound healing
Source: Br J Pharmacol. 2022 Sep 2;179(23):5222–32. doi: 10.1111/bph.15932 (PMC9826027; doi:10.1111/bph.15932)
Supplement: Supplementary file 2 — Table S1. The sequences of quantitative RT‐PCR primers [file BPH-179-5222-s002.docx]

**SUPPLEMENTARY FIGURES**

**Table S1. The sequences of quantitative RT-PCR primers**

| **Primers** | Forward | Reverse |
| --- | --- | --- |
| **IL6** | CTCTGGGAAATCGTGGAAATG | AAGTGCATCATCGTTGTTCATACA |
| **TNF-α** | GCCTCTTCTCATTCCTGCTTG | CTGATGAGAGGGAGGCCATT |
| **MCP-1** | ATCCCAATGAGTAGGCTGGAGAGC | CAGAAGTGCTTGAGGTGGTTGTG |
| **IL-10** | AGCCGGGAAGACAATAACTG | CATTTCCGATAAGGCTTGG |
| **Fizz1** | TCCCAGTGAATACTGATGAGA | CCACTCTGGATCTCCCAAGA |
| **Rantes** | GCCCTCACCATCATCCTCACT | GGCGGTTCCTTCGAGTGACA |
| **Fizz-1** | TCCCAGTGAATACTGATGAGA | CCACTCTGGATCTCCCAAGA |
| **Arg-1** | CGCCTTTCTCAAAAGGACAG | CCAGCTCTTCATTGGCTTTC |
| **CD31** | ATGACCCAGCAACATTCACA | TGCACAGGATGGAAATCACA |
| **Vegfa** | CAGGCTGCTGTAACGATGAA | AATGCTTTCTCCGCTCTGAA |
| **Fgf2** | CACCAGGCCACTTCAAGGAC | ATAGCAAGGTACCGGTTGGC |
| **Cxcl12** | TGCATCAGTGACGGTAAACCA | TTCTTCAGCCGTGCAACAATC |
| **Lcn2** | CTACAATGTCACCTCCATCCTGG | GCATATTTCCCAGAGTGAACTGGC |
| **Angio-2** | TAGCATCAGCCAACCAGGA | AAGGACCACATGCGTCAAAC |
| **VEGFR2** | ATCTTCAAGCCGTCCTGTGT | GCATTCCACATCTGCTGTGCT |
